# Analytical Validation of a New Immunoenzymatic Method for the Measurement of Feline Parathyroid Hormone in Cats with Chronic Kidney Disease

**DOI:** 10.3390/ani11113100

**Published:** 2021-10-29

**Authors:** Jari Zambarbieri, Pierangelo Moretti, Alessia Giordano, Paola Scarpa

**Affiliations:** Department of Veterinary Medicine, University of Milan, Via dell’Università 6, 16900 Lodi, Italy; pierangelo.moretti@unimi.it (P.M.); alessia.giordano@unimi.it (A.G.); paola.scarpa@unimi.it (P.S.)

**Keywords:** parathyroid hormone, cat, validation

## Abstract

**Simple Summary:**

Parathyroid hormone (PTH) is involved in many metabolic diseases, such as chronic kidney disease (CKD) and calcium disorders, and its measurement could be of clinical utility. However, available methods for the measurement of feline PTH are limited and not widely accessible. The aim of this study was to perform the analytical validation of a new method for PTH measurement in cats. Thirty-eight cats affected with CKD were included. The analytical protocol provided an evaluation of the precision, accuracy, and storage stability at different temperatures. The method investigated showed good precision and accuracy and good stability for 1 week of storage at freezing temperatures. The method was validated in cats, allowing its future use in diagnostic procedures.

**Abstract:**

The determination of parathyroid hormone (PTH) in cats could be of clinical utility in many metabolic disorders, such as renal diseases, hypercalcemia, or nutritional imbalances. However, the available methods for the measurement of feline PTH are limited, not widely available, and need radioimmunoassays. The aim of this study was to perform the analytical validation of a new immunoenzymatic method for the measurement of feline PTH. Thirty-eight cats affected with chronic kidney disease (CKD) were included. PTH was measured using a two-site immunoenzymatic method validated in humans and dogs (ST AIA-PACK^®^ Intact PTH, Tosoh Bioscience, Tessenderlo, Belgium). The analytical validation provided the evaluation of precision (intra-assay and inter-assay), accuracy (linearity under dilution (LUD) and spike recovery test (SRT)), and the storage stability of serum samples at 20 °C, 4 °C, and −20 °C. The method showed good precision (intra-assay CVs (coefficient of variations) 3.19–9.61%; inter-assay CVs 9.26–15.28%). In both the intra- and inter-assays, the highest imprecision was found with the low concentration pool (9.61% and 15.28%) and accuracy (LUD and SRT r^2^ = 0.99, *p* < 0.001), while the stability was optimal up until 7 days at −20 °C (−7.7%). The method was successfully validated in cats, allowing its future use in diagnostic procedures.

## 1. Introduction

Parathyroid hormone (PTH) is a single-chain, 84-amino-acid polypeptide produced by chief cells in the parathyroid glands and highly conserved among mammalian species; feline PTH is more than 83% identical to the canine and human molecules [1].

PTH is the main regulator of ionized calcium concentration through its direct effects on renal tubular reabsorption and bone resorption of calcium and indirect intestinal absorption of calcium mediated by calcitriol or active vitamin D. PTH also has a role in phosphorus metabolism, increasing bone resorption and decreasing reabsorption in renal tubules [2].

PTH is involved in many pathological conditions in which calcium and phosphorus metabolism is imbalanced; among these, primary hyperparathyroidism, renal secondary hyperparathyroidism (RHPT), nutritional secondary hyperparathyroidism, and hypercalcemia of malignancy. Specifically, RHPT is secondary to chronic kidney disease (CKD), a common disease in feline medicine affecting especially old cats (30–40% over 10 years) due to different underlying aetiologies; CKD induces many metabolic disorders involving calcium and phosphorus metabolism, resulting in hypersecretion of PTH [3].

The determination of PTH concentrations is therefore a fundamental tool in the diagnosis of calcium metabolism disturbances, in monitoring the treatment response, and in the evaluation of prognosis. In this context, routine measurement of PTH improves the ability to diagnose parathyroid disorders [1,4]. However, the currently available validated methods for the measurement of feline PTH are limited and not widely available.

To date, different generations of assays for the measurement of PTH in people and veterinary species have been reported. Briefly, first-generation PTH immunoassays used a single polyclonal antibody directed against the PTH C-terminal part or midterminal part. The finding of many biologically inactive C-terminal fragments detected by these assays led to the development of more specific second-generation assays using two antibodies directed at two different regions of the molecule. However, also these assays were found to measure some C-terminal fragments and a third-generation radioimmunoassay, able to detect specifically the full-length PTH of all 84 amino acids, has been recently developed [5].

In human medicine, an immunochromatographic test strip method able to detect tissue PTH for intraoperative parathyroid identification is also currently available, without current application in veterinary medicine [6].

Specifically, for PTH measurement in cats, a second-generation validated method (Allegro Intact PTH, Nichols Institute Diagnostics, San Juan Capistrano, CA, USA) is no longer available and only a third-generation assay (Duo PTH kit, Scantibodies Laboratory, Inc., Santee, CA, USA; total intact PTH IRMA-coated bead version, Part number 3KG600, Scantibodies Laboratory, Inc., Santee, CA, USA) is currently available, with the validation data reported by different authors [7,8,9]. However, also this method has several limitations, and it also has now been discontinued. First, radioimmunoassays are not available in all veterinary laboratories due to the high costs of the equipment and reagents needed. Moreover, the assays evaluated by Pineda et al. and Williams et al. showed a lower limit of detection of 3 pg/mL and 5.2 pg/mL, respectively, higher than the lower reference limit for cats [8,9]. Considering this scenario in which radioimmunoassays are discontinued, the possibility to expand the range of validated methods for the routine measurement of feline PTH would be useful.

The aim of this study was the analytical validation of a new immunoenzymatic method, already validated in humans and dogs, for the measurement of the feline PTH.

## 2. Materials and Methods

### 2.1. Animals and Samples

This study was carried out including 57 serum samples obtained from 38 cats affected with chronic kidney disease referred to the University of Milan Veterinary Teaching Hospital during routine clinical activity between February 2020 and April 2021. Fifteen cats were sampled more than once during the monitoring of the disease, each cat being sampled from 1 to 4 times.

As the severity of renal hyperparathyroidism is assumed to increase with the degree of azotaemia, cats at different CKD stages were included in the study, to increase the probability of obtaining samples with different PTH concentrations [10]. For all cats, the diagnosis and staging of CKD were performed according to the International Renal Interest Society (IRIS) guidelines: serum creatinine (sCr) persistently above 1.6 mg/dL and/or presence of renal proteinuria (urinary protein:creatinine ratio higher than 0.4) and/or presence of ultrasonographic abnormalities compatible with CKD. All cats included in the study were staged according to the IRIS guidelines [11].

All cats underwent a physical examination according to standard veterinary procedures and blood withdrawal was performed for diagnostics purposes after the owner’s consent. According to the Ethics Committee of the University of Milan (EC decision 29 October 2012, renewed with the protocol n° 02-2016), biological samples collected in this setting could also be used for research purposes and additional approval was not required. In total, 2 to 3 mL of blood were collected from the cephalic or jugular vein and placed into methacrylate tubes without anticoagulants, pre-filled with a gel separator and clot activator (FL Medical, Torreglia, Padua, Italy), followed by centrifugation (10 min, 2500× *g*) within 30 min from collection, and execution of routine analyses (including sCr) within 2 h. The leftover serum was utilized for the measurement of PTH.

### 2.2. PTH Measurement

The measurement of PTH was performed after routine analyses using an automated analyser (AIA 360^®^) and a two-site immunoenzymometric assay (ST AIA-PACK^®^ Intact PTH, Tosoh Bioscience, Tessenderlo, Belgium) validated in humans and dogs [12]. Briefly, the analysis is performed entirely in the test cups according to the procedure described. Intact PTH present in the sample is bound with a polyclonal antibody immobilized on the magnetic solid phase and an enzyme-labelled polyclonal antibody. The magnetic beads were washed to remove the unbound enzyme-labelled polyclonal antibody and were then incubated with the fluorogenic substrate 4-methylumbelliferyl phosphate (4MUP). The amount of enzyme-labelled polyclonal antibody that binds to the beads is directly proportional to the intact PTH concentration in the sample. The standard curve was constructed using six calibrators, provided by the manufacturer, with increasing concentrations of PTH: 0 pg/mL, 15.3 pg/mL, 48.3 pg/mL, 198 pg/mL, 770 pg/mL, and 2280 pg/mL. Three levels of control sera provided by the manufacturer, with concentrations of 10.70, 33.60, and 213.50 pg/mL, were run before each work session.

### 2.3. Analytical Validation

The analytical validation was performed using the calibrators, control materials, and reagents provided by the manufacturer, which are all human-based. All the pooled sera were obtained by merging specimens from cats included in the study and grouped based on their PTH concentration, following preliminary assessment through measurement on single samples.

The intra-assay imprecision was determined by measuring PTH in feline pooled sera with low (3.65 pg/mL), medium (24.12 pg/mL), and high (102.90 pg/mL) PTH levels; 5 replicates of each measurement within a single run of analysis were done on each pool. The inter-assay variability was assessed by analysing the same samples, in duplicate, on 5 consecutive working days. The mean value, standard deviation (SD), and coefficient of variation (CV = SD/mean × 100) were calculated.

The accuracy was determined by evaluating the linearity under dilution (LUD) and the spike-recovery test (SRT): LUD was performed by measuring PTH on a pool of feline sera with a high PTH concentration (86.5 pg/mL) after serial dilutions with a 0.9% NaCl saline solution to obtain solutions containing 90%, 80%, 70%, 60%, 50%, 40%, 30%, 20%, 10%, 5%, 2.50%, and 1.25% of the serum sample, respectively. In the absence of an accepted gold standard to measure canine PTH, nor any available purified feline PTH, SRT was performed by mixing a pool with a low PTH level (1.1 pg/mL) with increasing percentages (10% to 100%) of a pool with a medium PTH level (14.1 pg/mL). The association between the obtained and the expected values in the LUD and SRT tests was assessed using a least-squares regression analysis performed by the statistical software JMP 16 (SAS Inc, Cary, NC, USA).

The lower limit of detection (LLOD) of the method was obtained using the following formula: LLOD = blank + 1.645 (SD_low concentration sample_), where blank is the assay sensitivity provided by the manufacturer (1 pg/mL) and the low concentration sample is the mean of the lowest values obtained by LUD [13]. The LLOD was calculated with this method because in the measurement of the zero calibrator or blank the instrument does not provide numerical results.

### 2.4. Storage Stability

Storage stability was evaluated using a pool of fresh feline sera with a mean of 11.05 pg/mL, analysed in duplicate immediately after sampling and then split in different aliquots and stored under different temperatures: room temperature (about 20 °C), refrigeration temperature (4 °C), and freezing temperature (−20 °C). The analysis was repeated in duplicate after the following times: 6 h (within the same work shift) and 24 h (between a work shift and the following) at room temperature; 6 h, 24 h, 48 h, and 72 h at refrigeration temperature; and 1 week and 1 month at freezing temperature. A deviation from the baseline of ±10% was considered tolerable, as reported by previous validation studies [14,15].

## 3. Results

The population was composed of 34 Domestic Shorthairs, 1 American Curl, 1 Birman, 1 Bengal, and 1 Maine Coon; the age ranged from 1 to 19 years with a median of 10 years; according to sex, 18 neutered males, 16 neutered females, 3 entire males, and 1 entire female were present.

The CKD staging according to the IRIS staging system at first examination was as follows: 1 cat was classified in Stage 1, 26 in Stage 2, 4 in Stage 3, and 7 in Stage 4.

Serum creatinine ranged from 1.5 to 16.4 mg/dL, with a median of 2.4 mg/dL.

Serum PTH ranged from 1.3 (excluding values below the LLOD) to 377.6 pg/mL, with a median of 11.9 pg/mL. According to the IRIS stage, the only cat in Stage 1 had a concentration of PTH of 11 pg/mL; the median PTH values were 9.8 (1.3–362) pg/mL in Stage 2; 18.9 (1.4–130.1) pg/mL in Stage 3; and 94.8 (3.9–377.6) pg/mL in Stage 4. In nine cats, the PTH value was lower than the limit of detection of the instrument; these samples were used in the low pools.

The complete data are reported in Supplementary Materials Table S1.

### 3.1. Precision and Accuracy

The results of the intra- and inter-assay precision assessment evaluated on the pooled sera at low, medium, and high PTH concentrations are reported in Table 1. The intra-assay CVs were <10% at the three levels of PTH concentration; the inter-assay CVs were between 9.26% and 15.28%. In both the intra- and inter-assays, the highest imprecision was found with the low concentration pool (9.61% and 15.28%, respectively).

**Figure 1:**
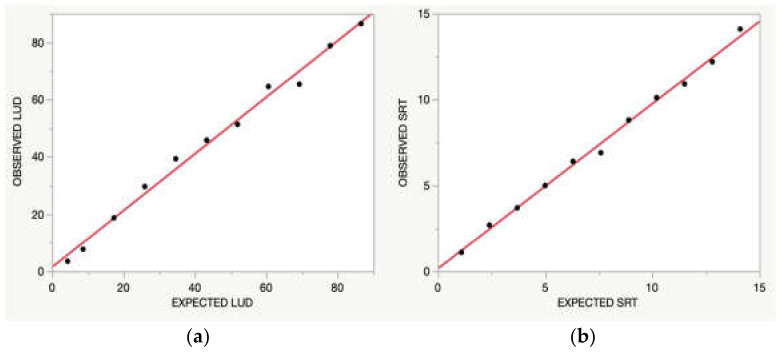
Comparison between the observed and expected results: (**a**) linearity under dilution (LUD) of the PTH concentration in a pool of sera serially diluted with saline solution; (**b**) spike recovery test (SRT) of the PTH concentration in a pool of sera with a low PTH concentration spiked with increasing amounts of a serum with a medium PTH concentration. The solid line indicates the linear association between the expected and observed results.

The results obtained after LUD and SRT are reported in Figure 1. Both tests fitted the linear model (r^2^ = 0.99, *p* < 0.001 for the LUD test; r^2^ = 0.99, *p* < 0.001 for the SRT), showing a satisfying agreement between the observed and expected results. This part of the study allowed the determination of the LLOD of the method, which was about 1.1 pg/mL.

### 3.2. Storage Stability

Storage stability was evaluated using a pool of sera with a medium PTH concentration (11.05 pg/mL) tested at different storage conditions (Table 2). The deviation from baseline exceeded 10% after 6 h of storage at both 20 °C and 4 °C, ranging between 14.5% (6 h at 4 °C) and 51.1% (24 h at 20 °C). At freezing temperature (−20 °C), the deviation from the baseline was minimal until 7 days storage, whereas it exceeded 10% after 1-month storage.

## 4. Discussion

Feline hyperparathyroidism is a documented disease, either in its primary or secondary form, i.e., nutritional, and renal secondary hyperparathyroidism [16]. Specifically, the prevalence of RHPT is reported to be between 47% and 100% of cats affected with CKD (mean 84%), with a possible increased PTH concentration also in the early stages of the disease, before evidence of calcium and phosphorus imbalances [17,18]. It is also reported that the evaluation of PTH concentrations could help predict the development of azotaemia in geriatric normoazotemic cats [19]. According to these data, to achieve our aim, we included cats with azotemic CKD (except one in Stage 1) in which the PTH levels are often higher compared to healthy cats.

However, PTH measurement is not routinely performed due to the limited availability of methods specific for feline PTH, and the known instability in serum samples, as previously reported [7]. The current reference method for the determination of feline PTH is the expensive third-generation whole-PTH radioimmunoassay [16]. For these reasons, the validation of a new immunoenzymatic method able to detect feline PTH, avoiding the employment of a radioimmunoassay, would be useful.

In the present study, the ST-AIA PACK^®^ Intact PTH assay showed satisfying results in the detection of feline PTH in serum samples and could be used also on plasma samples, even if specific data about possible discrepancies are not available. Intra- and inter-assay CVs were within the limit of 15%, which is considered acceptable for an immunoassay [20], especially for hormones that are usually affected by elevated variability. Only the inter-assay CV with the low concentration pool was very slightly above the limit (15.3% vs. 15%); however, even if the diagnostic accuracy of this method has not been evaluated in other studies and was beyond the scope of the present study, such low concentrations are generally found only in healthy cats and thus the imprecision may be considered acceptable from a clinical point of view. Overall, these results are quite similar, and in some cases lower, compared with those reported in other validation studies, in which the CVs were approximately constantly near 10% [7,8,9,21], underlining that the investigated method shows acceptable analytical repeatability and reproducibility.

In absence of a current gold standard for the measurement of feline PTH, the indirect evaluation of accuracy using SRT and LUD showed an optimal concordance between the obtained and expected results, suggesting that this method can be considered reliable for PTH measurement also in cats.

Interestingly, the lower limit of detection identified, about 1.1 pg/mL, was lower than those obtained with other methods, in which LLOD was 2 pg/mL, 3 pg/mL, and 5.2 pg/mL [8,9,22]. This analytical sensitivity will allow to correctly measure the PTH concentration in healthy cats and in cats with early stages of CKD, in which RHPT may be absent.

The evaluation of storage stability was done to define the best storage condition that allows accurate measurements, considering that only limited and not recent data about feline PTH stability are available. Barber et al. reported good stability at −20 °C up until 30 days and suggested the freezing of the sample within 2 h of collection [7]. In our study, at room and refrigeration temperatures the deviation from the baseline exceeded 10%, which was established as a cut-off of tolerance, already after 6 h, leading to lower PTH values. This decrease was even more evident after 24 h, discouraging the use of long-time stored samples for PTH measurement in cats. This observation has two possible explanations: firstly, the proteolytic degradation of the molecule could decrease the serum PTH concentration, as reported for other hormones such as adrenocorticotropic hormone [23]; secondly, the high deviation from the baseline could be attributable to the relatively low concentration of PTH (11.05 pg/mL) in the sera used for the evaluation of storage stability. However, the decrease of 14.5% after 6 h at 4 °C and about 20% after 6 h at 20 °C and after 24 h at 4 °C could be considered acceptable for clinical purposes, especially in cats affected with CKD in which the PTH concentrations are significantly higher [19]. The good stability observed for 7 days at −20 °C suggests freezing the samples as soon as possible after collection and storing at freezing temperature before PTH measurement, as already suggested and required by some laboratories for other hormones [7].

## 5. Conclusions

This study demonstrates that the ST-AIA Pack Intact PTH is a precise and accurate immunoenzymometric assay for the detection of PTH in feline serum and, considering the very limited availability of assays for the measurement of feline PTH, this could be relevant in clinical practice for the diagnosis of feline primary and secondary hyperparathyroidism.

The storage stability is limited but compatible with working turnaround times as determinations should be performed on the same day of sampling when stored at room temperature or refrigerated but can be performed within one week when stored at freezing temperature.

## Figures and Tables

**Table 1:** Intra- and inter-assay imprecision calculated using pooled sera with a low, medium, and high parathyroid hormone concentration (pg/mL). SD: standard deviation; CV: coefficient of variation.

Parameter	Low	Medium	High
**Intra-Assay**			
Mean	3.65	24.12	102.90
SD	0.35	1.17	3.28
CV	9.61	4.85	3.19
**Inter-Assay**			
Mean	3.07	21.21	92.46
SD	0.47	2.26	8.56
CV	15.28	10.63	9.26

**Table 2:** Deviation from the baseline concentration of PTH (pg/mL) expressed in % at different storage temperatures. h: hours; d: days; n.d.: not done.

Time Interval	Storage Temperature		
	20 °C	4 °C	−20 °C
T 0	11.05	11.05	11.05
6 h	8.75 (−20.8%)	9.45 (−14.5%)	n.d.
24 h	5.4 (−51.1%)	8.7 (−21.3%)	n.d.
7 d	n.d.	n.d.	10.2 (−7.7%)
30 d	n.d.	n.d.	8.45 (−23.5%)

## Data Availability

The data presented in the study are available on reasonable request from the corresponding author.

## Supplementary Materials

The following are available online at https://www.mdpi.com/article/10.3390/ani11113100/s1, Table S1: Complete data of samples and animals included.
